# Upadacitinib for the treatment of geographic tongue: A case report

**DOI:** 10.1016/j.jdcr.2026.02.015

**Published:** 2026-02-13

**Authors:** Alexa Moschella, Mark G. Kirchhof

**Affiliations:** a Faculty of Medicine, University of Ottawa, Ottawa, Ontario, Canada; b Division of Dermatology, Department of Medicine, The Ottawa Hospital, Ottawa, Ontario, Canada

**Keywords:** benign migratory glossitis, geographic tongue, Janus kinase inhibitor, upadacitinib

## Introduction

Up to 3% of the population is affected by geographic tongue (GT), a relapsing and recurring inflammatory condition of unknown etiology.^1^^,^^2^ GT is characterized by atrophy of filiform papillae, typically on the dorsal and lateral surfaces of the tongue, which leaves irregular erythematous patches surrounded by raised cream-colored keratotic bands.^1^^,^^2^ Lesions are variable and dynamic, with morphology that can change within hours.^3^^,^^4^ While typically self-limiting, GT may persist for days to weeks and can recur unpredictably at different sites with different morphology.^3^^,^^5^^,^^6^ Although usually asymptomatic, symptomatic GT can be highly debilitating, producing pain, burning, foreign body sensation in the mouth, dysgeusia, and sensitivity to hot or spicy foods.^1^^,^^6^^,^^7^ Despite this, no definitive treatment has been established.^7^ Herein, we present a case of symptomatic GT treated with upadacitinib, a Janus kinase (JAK) inhibitor.

## Case report

**Fig 1:**
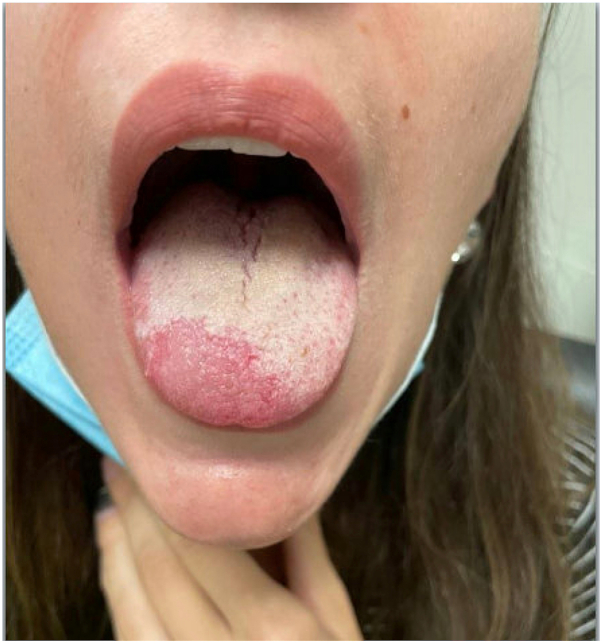
Initial presentation of a well-demarcated irregular erythematous patch on the right distal tongue surrounded by a raised *cream-colored* border.

A 30-year-old female presented to the emergency department with a 4-month history of erythema and burning sensation, which originated on the posterior but migrated to the distal tongue. It had acutely worsened over the past 6 weeks causing irritation with food and water intake. She initially noticed tongue lesions following a viral upper respiratory tract infection, which improved minimally before recurring. She also reported a 6-week history of a productive cough. One week prior to the presentation, she was prescribed ciclesonide nasal spray and amoxicillin for sinusitis. When the patient was experiencing symptoms, vitamin D, B6, ferritin, and zinc were normal. Vitamin B12 was initially low (164 pmol/L, normal: >220 pmol/L) but recovered 1 month before presentation (828 pmol/L, normal: >220 pmol/L). On examination, a well-demarcated irregular erythematous patch on the right distal tongue surrounded by a raised cream-colored border was appreciated (Fig 1). She was diagnosed with GT and an asthma exacerbation and was counseled on the self-limiting nature of GT without further treatment.

**Fig 2:**
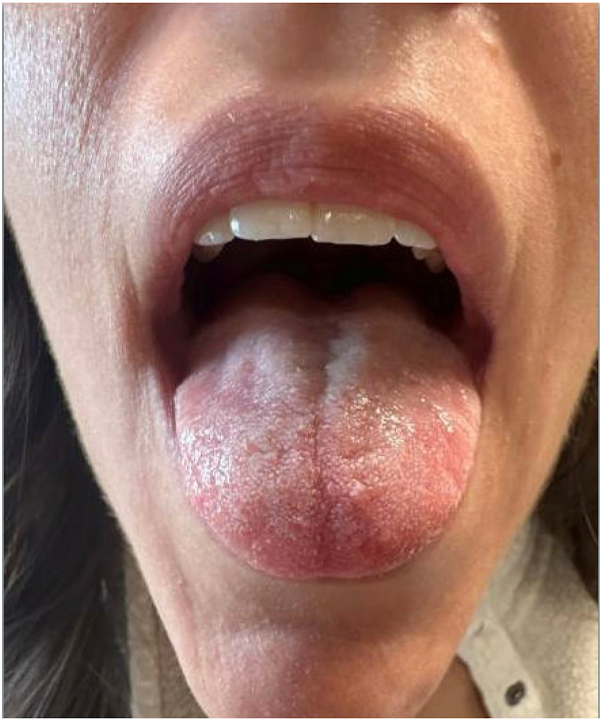
Uniform tongue coloration without visible lesions after 8 months of treatment with upadacitinib.

The patient re-presented to the emergency department 7 weeks later with a painful, burning white plaque on the dorsal tongue, which intermittently worsened (thickened) and improved. She was treated with nystatin and oral fluconazole by her family physician without improvement. She reported tongue swelling, worsening pain requiring ibuprofen, and reduced ability to tolerate oral intake. She denied odynophagia, dysphagia, voice changes, chest pain, or dental concerns. The patient was a nonsmoker, used minimal alcohol, and had no known allergies or relevant family history. Her only medication was levonorgestrel-ethinyl estrad 100-20 mcg tablets. On physical exam, a nonscrapable, confluent white plaque on the dorsal tongue with white papillae was appreciated. No other oropharyngeal or extraoral lesions were present. She was referred to ear, nose, and throat, where a biopsy was performed during a flare, which revealed nonspecific acanthotic squamous epithelium with mild perivascular infiltrate. Direct immunofluorescence was negative. Postbiopsy, she developed a severe flare of tongue lesions and a new rash on the arms and torso. She reported that the rash was treated as psoriasis by her family physician and resolved with a corticosteroid. At ear, nose, and throat follow-up, she was prescribed triamcinolone acetonide 0.1% in orabase as needed, which minimally reduced erythema during flares, and a 10-day course of prednisolone sodium phosphate 5 mg/mL oral solution, which had no effect. She was then referred to dermatology, where she reported developing new lesions a few times per month, which persistently affected the anterior tongue and resolved within days to weeks. Upper respiratory tract infections, acidic foods, and spicy foods were identified as triggers. The pain and burning sensation progressively worsened her ability to eat. On physical examination, 2 well-demarcated areas of enlarged papilla with no involvement of the buccal mucosa or gingiva were noted. Given the need for long-term treatment due to nearly 2 years of recurrent, severe symptoms refractory to topical management, oral upadacitinib 15 mg once daily was prescribed. Oral contraception was later discontinued. At the 6-week follow-up, she reported a 40% to 50% improvement in symptoms. At 8 months, she noted occasional flares with upper respiratory tract infections, acidic foods, and spicy foods, but with reduced burning and fewer lesions. Uniform tongue coloration without visible lesions was appreciated on examination (Fig 2). At 10 months, she continues to be satisfied with treatment, her only reported side effect being increased frequency of viral illness which she considers tolerable.

## Discussion

Similar to our patient’s demographic profile, GT is most prevalent in patients 20 to 29 years of age with a slight female predominance.^6^ Despite unclear etiology, studies have shown a link between asthma, eczema, allergies, and GT, suggesting a pathogenesis similar to atopy.^5^^,^^6^ GT has also been associated with psoriasis^3^^,^^6^ and its severity has been implicated with oral contraceptive use.^1^^,^^8^ Additional proposed contributors include genetic factors, emotional stress, vitamin deficiency (vitamins D, B6, B12, folic acid, iron, and zinc), and infection.^6^^,^^9^ Our patient’s history of infection, asthma, oral contraceptive use, low B12, and suspected psoriasis may have increased susceptibility.

Treatment is warranted in persistent and severe cases. Topical steroids, retinoic acid, cyclosporine, antihistamines, and tacrolimus have been utilized for symptomatic management, yet none are specific nor curative.^5^^,^^9^ A randomized controlled trial found that the application of 0.1% triamcinolone acetonide in an oral base with or without 0.05% retinoic acid twice daily for 10 days reduced pain and burning sensation, with smaller lesions after treatment.^9^ Although our patient utilized this treatment as needed, it failed to provide substantial relief.

To our knowledge, this is the first reported case of GT treated with a JAK inhibitor. Upadacitinib is an oral selective JAK inhibitor that blocks cytokine signaling in inflammatory disease pathways.^10^ After failing multiple prior therapies, our patient experienced marked improvement in burning sensation, reduced number of lesions, and less severe flares following initiation of upadacitinib.

Upper respiratory tract infections have been reported as adverse events associated with upadacitinib^10^; however, the benefits of GT control may outweigh mild adverse events. Our patient found frequent infection tolerable given the control of her GT.

Overall, this case highlights therapeutic potential for JAK inhibitors in the treatment of symptomatic recalcitrant GT. Further studies are required to assess long-term safety, efficacy, and recurrence rates of GT treated with JAK inhibitors.

## Conflicts of interest

None disclosed.
